# Peripheral Neutrophil to Lymphocyte Ratio (NLR), a cogent clinical adjunct for Ki-67 in breast cancer

**DOI:** 10.1186/s43046-023-00200-4

**Published:** 2023-12-25

**Authors:** Radhika Arora, Feroz Alam, Atia Zaka-ur-Rab, Veena Maheshwari, Kiran Alam, Mahboob Hasan

**Affiliations:** 1 Department of Pathology, Jawaharlal Nehru Medical College, Aligarh Muslim University, Aligarh, 202002 U.P India; 2 Department of General Surgery, Jawaharlal Nehru Medical College, Aligarh Muslim University, Aligarh, India

**Keywords:** Ki-67, NLR, Breast cancer, Prognosis, Personalized biomarker

## Abstract

**Background:**

Clinical utility of Ki-67 immunohistochemistry (IHC) in breast cancer (BC) is mainly limited to decide for the use of chemotherapy and estimate prognosis in patients with either Ki-67 index < 5% or > 30%; however, lacunae still exists pertaining to its analytical validity. Neutrophilia is common in cancer with accompanying lymphocytopenia. Neutrophil to lymphocyte ratio (NLR) captures the intricate balance between pro-tumor neutrophilia and anti-tumor lymphocyte immunity. This study aimed to correlate cellular proliferation in breast cancer with NLR.

**Methods:**

An observational study was carried out including 73 cases of BC; pre-treatment NLR and Ki-67 grading were performed. NLR < 3 was considered low, while ≥ 3 was high. The Ki-67 expression was graded as low ≤ 5%, intermediate 6–29%, or high ≥ 30%. Various clinico-pathological variables were studied, and the association of categorical variables was analyzed using Pearson’s chi-square test, and a *p*-value of < 0.05 was taken as significant.

**Results:**

Ki-67 correlated significantly with modified Scarff-Bloom-Richardson (SBR) grade (*p* < 0.01), and tumor-node-metastasis (TNM) stage (*p* < 0.001). Correlation of NLR was not significant with SBR grade (*p* > 0.05) and molecular subtype (*p* > 0.05); however, NLR was found to be significantly correlated with TNM stage (*p* < 0.001) and Ki-67 (*p* < 0.001).

**Conclusion:**

NLR is fast emerging as a personalized theranostic marker in breast cancer. Instead of determining a generalized cut-off value, individual baseline NLR and its dynamics with disease progression will help manage patients better, obviating some of the drawbacks associated with Ki-67.

## Background

Ki-67 expression is a reliable predictor of proliferative activity of the tumor cells in colorectal, prostate, gastric, and other cancer types [1–4]. In addition, prognostic significance of Ki-67 expression has been documented, and presently systemic therapeutic strategies are being deduced from the expression levels of Ki-67 in certain malignancies [5]. In BC, Ki-67could signify responsiveness/ resistance to chemotherapy /endocrine therapy [6], estimate the residual risk in patients on standard therapy, and can also predict treatment effectiveness before, during, and after neoadjuvant therapy, especially neoadjuvant endocrine therapy [7, 8]. The 12th St. Gallen Expert Consensus panel in 2011 established that a Ki-67 expression < 14% in Estrogen Receptor (ER) positive, Human Epidermal Growth Factor Receptor 2 (Her 2/neu) negative patient represents the luminal A subtype, while ER positive Her 2 negative patients with Ki-67 expression > 14% represents the luminal B subtype [9]. However, in 2013, the St. Gallen consensus statement re-defined Ki-67 expression > 20% as the new threshold for classifying breast cancer luminal subtypes [10], since tumors with a higher Ki-67 expression were more likely to get benefitted from cytotoxic chemotherapy. Meanwhile, it was also seen that Ki-67 immuno-expression suffered from low reproducibility of results, principally in the intermediate immuno-expression group (ranging from 15 to 30%). So, in the last Saint Gallen Conference (March 2015), the consensus cut-off for Ki-67 had to be specifically made in the light of local laboratory reports [11].

Triple-negative breast cancer (TNBC) is an aggressive malignancy with poor prognosis and does not benefit from either targeted therapy or endocrine therapy [12]. Newer biomarkers are continuously being searched for better patient management; Ki-67 IHC is also being investigated as a possible predictive and prognostic biomarker following neoadjuvant chemotherapy (NAC) in patients with TNBC [13]. Generally, TNBC shows a higher baseline Ki-67 immuno-expression than the luminal tumors. A meta-analysis of 35 studies with 7716 enrolled patients concluded that a high Ki-67 of ≥ 40% is associated with a greater risk of recurrence and death compared with lower expression rates [14]. Therefore, currently, the role of Ki-67 in therapeutic decision making still remains controversial, be it luminal A, luminal B, or the triple negative variant. Furthermore, inconsistencies in Ki-67 IHC are reported at the pre-analytical, analytical, interpretation, as well as the data analysis stages [15]. To standardize Ki-67 scoring, the International Ki-67 in Breast Cancer Working Group (IKWG) was established in 2011 [8]; in its last meeting held in October 2019, the IKWG recommended the following: (1) Pre-analytical handling considerations are critical in Ki-67 scoring. (2) A standardized visual method for estimating Ki-67 has to be adopted. (3) Analytical validity of Ki-67 score must be maintained by quality assurance and quality control programs. (4) Lastly, Ki-67 IHC identifies T1-2 and N0-1, ER + / HER2 − breast cancer patients who do not need (≤ 5% score) or need (≥ 30% score) adjuvant chemotherapy and thence signifies prognosis [16].

NLR is being increasingly used as a reliable and easy available marker of immune response to various infectious and non-infectious stimuli. Normally, NLR lies between 1 and 2; the values higher than 3.0 and below 0.7 in adults are definitely abnormal. The intermediate NLR (2.3–3.0) may serve as an early warning of the varied pathological processes like cancer, atherosclerosis, infection, inflammation, psychiatric disorders, and stress. NLR is used as a reliable and cheap marker of prognosis in solid tumors. Meta-analyses explored the prognostic significance of NLR in solid tumors and found a cut-off value of NLR above 3.0 [17]. The negative impact of elevated NLR on BC patient’s outcome has been documented [18] including the TNBC subtype [19]. During an inflammatory process (infective/allergic/tumoral), the hematopoietic stem cells (HSCs) in the bone marrow detect peripheral perturbations through danger signals [20]. Confronting the stress, HSCs undergo a myeloid-biased differentiation mediated via cytokines; this increased output of myeloid cells is called stress-induced myelopoiesis or emergency myelopoiesis [21]. This emergency myelopoiesis may have a role in host immunity, promoting disease development and progression [20]. The release of these stress-induced myeloid cells into the peripheral circulation is usually reflected as a raised NLR.

The present study aims to unravel any link between tumor proliferative potential (Ki-67 score) and tumor-induced emergency myelopoiesis (NLR).

## Methods

### Study outline

An observational study was carried out in the Department of Pathology in collaboration with the Department of Surgery, J.N Medical College, Aligarh Muslim University, India, from November 2019 to November 2021. Seventy-three consenting female patients with core needle biopsy-based newly diagnosed, early, or locally advanced BC were enrolled for the study. All the necessary details about the process of the study and the rationale behind the study were explicitly explained to the patients in their own language, and any doubts were clarified. Patients refusing consent, those who received any prior neoadjuvant chemotherapy and/ or radiotherapy, with known metastatic lesions or inflammatory subtype of breast cancer, were excluded from this study. NLR of study participants was noted prior to any surgical/ therapeutic intervention (even before performing the core needle biopsy). To calculate NLR, 2 ml peripheral venous blood was collected in Ethylenediaminetetraacetic acid (EDTA) anti-coagulant vial, and the cell counts were performed by Bene Sphera H33 3-part hematology analyzer. NLR was then calculated as a ratio of absolute neutrophil count to absolute lymphocyte count and was categorized as low < 3 or high ≥ 3. After the core needle primary Hematoxylin & Eosin (H&E) based histopathological diagnosis, the Ki-67 IHC was performed. The primary antibody used was Thermo Scientific Ki-67 purified rabbit polyclonal antibody, and the secondary antibody used was Horse Radish Peroxidase (HRP). Ki-67 IHC was performed by the fully automated slide preparation system, Ventana Benchmark GX. The slides were then examined under a light microscope (Magnüs MX21i LED) using 100 × and 400 × magnification. For routine IHC reporting of BC patients, a Ki-67 cut-off of 14% was used to differentiate luminal A from luminal B [9]. However, for the purpose of this study, the Ki-67 was classified using the guidelines as recommended by the IKWG 2019 consensus meeting. Finally, the expression of Ki-67 was graded as low ≤ 5%, intermediate 6–29%, and high ≥ 30% [16]. Clinical data was collected from the hospital archives, and pathological observational data was collected and recorded electronically.

### Statistical analysis

The statistical analysis was done using Statistical Package for the Social Sciences (SPSS) version 25.0, categorical data were expressed as frequencies and continuous data as mean ± standard deviation, and the association of categorical variables was analyzed using Pearson’s chi-square test. A *p*-value of < 0.05 was taken as significant.

## Results

In our study, 32.9% of patients were ≤ 40 years of age, while 67.1% of patients were > 40 years of age. In the context of menopausal status, 42.5% of females were pre-menopausal, while 57.5% of them were menopausal. All the patients had a presenting complaint of a breast mass; in addition to it, 56.1% had concomitant axillary lymphadenopathy, 19.1% had fungating superficial ulcer, 17.8% had pain, and 7.0% had nipple discharge/retraction. The right side breast was involved in 56.2% of the cases, while the left side breast was involved in 43.8% of the cases. Location-wise, the upper inner quadrant was involved in 12.3% of the cases, and the upper outer quadrant was the most commonly affected accounting for 46.6% of the cases. The lower quadrant was less commonly involved with 2.7% of the cases in its outer part and 4.1% in its inner part. The central portion of the breast was involved in 15.1% of the patients and in 19.2% of the patients multiple quadrants of the breast were involved. Microscopic examination of the routine H&E stained slides identified that invasive breast cancer (IBC) of no special type (NST) was the commonest histologic variant with 74.0% of the cases, followed by IBC with medullary features 12.3%, metaplastic breast carcinoma 5.5%, and others 8.2% (mucinous carcinoma, invasive carcinoma with neuroendocrine differentiation, encapsulated papillary variant, etc.). The clinico-pathologic characteristics of the cases have been summarized in Table 1.

**Table 1:** Distribution of clinico-pathologic characteristics of study cases

Characteristic	No. of cases (total = 73)	%
1. *Age (years)*		
≤ 40	24	32.9
> 40	49	67.1
2. *Menopausal status*		
Pre-menopausal	31	42.5
Post menopausal	42	57.5
3. *Presenting complaints*		
Breast lump	73	100
Lump + Axillary lymphadenopathy	41	56.1
Lump + Fungating ulcer	14	19.1
Lump + Pain	13	17.8
Lump + others	05	7.0
4. *Laterality*		
Right	41	56.2
Left	32	43.8
5. *Location*		
Upper inner quadrant	9	12.3
Upper outer quadrant	34	46.6
Lower outer quadrant	2	2.7
Lower inner quadrant	3	4.1
Central	11	15.1
Multiple	14	19.2
6. *Histological type*		
Invasive Breast Cancer (IBC) NST	54	74.0
IBC with medullary features	9	12.3
Metaplastic breast carcinoma	4	5.5
Others	6	8.2

**Fig. 1:**
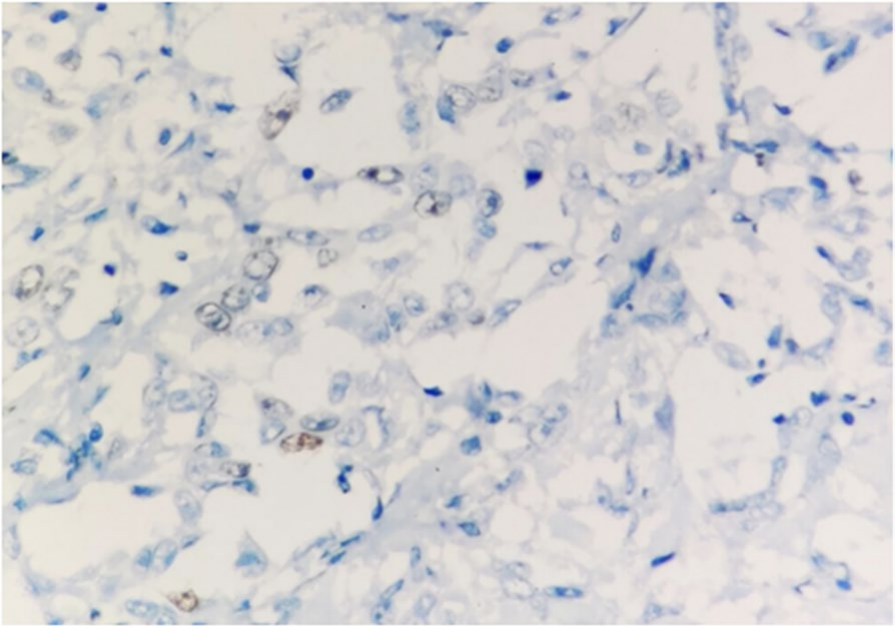
Tumor with low-grade Ki-67 expression < 5% (IHC × 400)

**Fig. 2:**
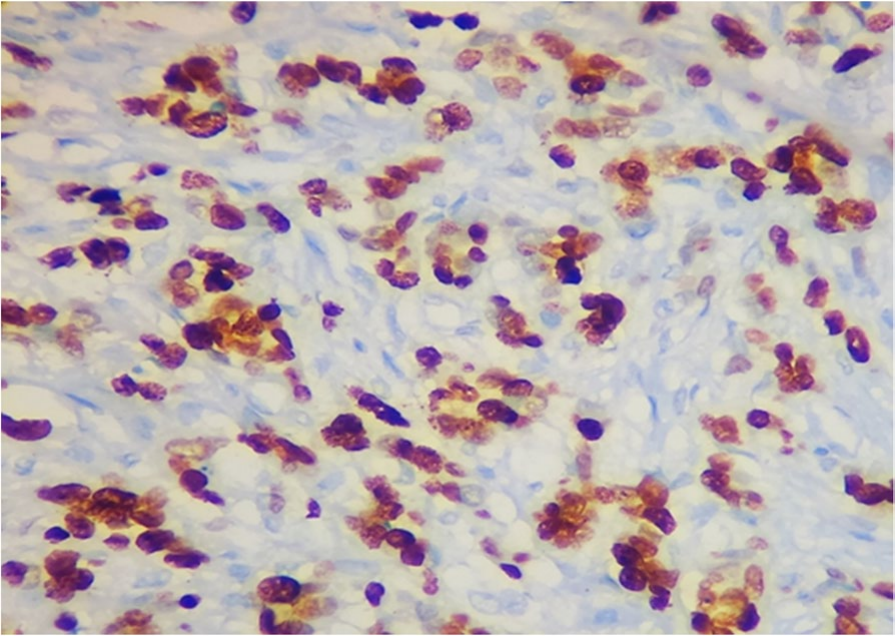
Highly proliferating tumor with high-grade Ki-67 expression > 30% (IHC × 400)

TNM staging of the cases showed that none of the case presented in stage I, 58.9% of patients were in stage II at the time of presentation, and 41.1% of patients were in stage III. Patients with distant metastasis were excluded from this study as mentioned earlier. The modified SBR grading of the cases was undertaken, and 11% of cases were of grade 1, 76.8% of grade 2, and 12.2% were of grade 3. Molecular sub-typing of all the cases was done by ER, PR, Her 2 Neu, and Ki-67 IHC as a standard procedure. Upon interpretation of the IHC results, luminal A was present in 20.5% of the cases, 19.2% of patients were of luminal B, 38.3% cases were of triple negative, and the remainder 22.0% of cases were of Her 2 enriched subtype. Subsequently, Ki-67 IHC classification was done, and it showed that 15.0% of the cases were of low grade (Fig. 1), 28.8% of intermediate grade, and 56.2% of high grade (Fig. 2). Similarly, NLR was calculated, and 64.4% cases had high (≥ 3) NLR, while 35.6% of the cases had low (< 3) NLR. On applying Pearson’s chi-square test, Ki-67 grade was significantly correlated with TNM stage of the tumor (Ch^2^-value 38.124, *p* < 0.001), and SBR grade (Ch^2^-value 16.595, *p* < 0.01). NLR showed a statistically significant correlation with TNM stage (Ch^2^-value 36.948, *p* < 0.001); however, NLR values were not found to be significantly correlated with either the SBR grade (Ch^2^-value 4.878, *p* > 0.05) or the molecular subtype of breast cancer (Ch^2^-value 4.856, *p* > 0.05). Lastly, the Ki-67 grade showed the strongest statistical correlation with NLR (Ch^2^-value 43.107, *p* < 0.001) as mentioned in Table 2.

**Table 2:** Correlation of clinico-pathologic variables and Ki-67 with NLR

Cases (*n* = 73)	**NLR**		Ch^2^-value	*p*-value
	Low (*n* = 26)	High (*n* = 47)		
**SBR grade**				
1 (*n* = 8)	5 (62.5%)	3 (37.5%)	4.878	> 0.05
2 (*n* = 56)	20 (35.7%)	36 (64.3%)		
3 (*n* = 9)	1 (11.1%)	8 (88.9%)		
**Molecular subtype**				
Luminal A (*n* = 15)	8 (53.3%)	7 (46.7%)	4.856	> 0.05
Luminal B (*n* = 14)	6 (42.9%)	8 (57.1%)		
Triple negative (*n* = 28)	6 (21.4%)	22 (78.6%)		
Her2 enriched (*n* = 16)	6 (37.5%)	10 (62.5%)		
**TNM stage**				
I (*n* = 0)	0	0	36.948	< 0.001
II (*n* = 43)	25 (58.1%)	18 (41.9%)		
III (*n* = 30)	1 (3.3%)	29 (96.7%)		
**Ki-67 expression**				
Low (*n* = 11)	11 (100%)	0	43.107	< 0.001
Intermediate (*n* = 21)	13 (61.9%)	08 (38.1%)		
High (*n* = 41)	02 (4.9%)	39 (95.1%)		

## Discussion

Proliferation at the cellular level decides the clinical behavior of BC; Ki-67 labeling index and mitotic count are known to identify cell proliferation in a tumor. Ki-67 labeling index seems to be a better guide of cell proliferation than mitotic figure counts as all the cells that are in active phases of the cell cycle can be recognized. Also, counting colored Ki-67 positive nuclei on IHC is easier than assessing mitotic figures [22, 23]. Ki-67 grade was higher in SBR grade 3 tumors as compared to grade 2 or and grade 1 tumors in our study. Similar results were discerned by Inwald et al. who reported an association between Ki-67 and tumor grade [24]. We found a significant correlation between Ki-67 and TNM stage (excluding metastatic/ stage IV) as demonstrated earlier by Kamranzadeh et al. [25] and Thangarajah et al. [26] that there is a significant positive correlation of Ki-67 index with clinical stage.

The TNM classification has conventionally been used as an indicator of cancer prognosis, but cancers at the same stage of progression vary in their prognosis because of the varied and unique host factors such as tumor microenvironment, nutritional status, and immune status among others. Recently, neutrophils are proven to affect tumor initiation, progression, and metastasis, and a raised NLR is emerging as a key indicator of cancer development and progression in several cancers, including breast cancer [27]. Its prognostic significance has been well defined in advanced tumors under the influence of tumoral cytokines and growth factors [28]. In our study, the statistical correlation between NLR and tumor SBR grade was not significant, and an extensive study conducted by Dirican et al. also showed similar results [29]. Different molecular subtypes of breast cancer also showed a non-significant correlation with NLR in our study. Earlier, it has been shown that no significant correlation is present between NLR and different molecular subtypes [30]. We found a significant correlation between NLR and tumor stage, similar to what has been documented by Elyasinia et al. in 2017 [31]. Few studies had already shown that NLR correlates well with Ki-67 expression in breast cancer. With a cut-off of 14%, Ki-67 was significantly correlated with NLR in the study of HER 2 negative breast cancer patients done by Bae et al. [32]. Low NLR along with platelet lymphocyte ratio (PLR) is an independent predictive factor for pathological complete response (pCR) in patients with early or locally advanced BC treated with NAC as this signifies a robust immune status [33]. Not only patient stratification and chemotherapeutic response, NLR is also associated with favorable prognosis in BC. Recently, NLR has been identified as an independent prognostic factor associated with better overall survival in oligometastatic BC [34]. Moving further with cancer progression, NLR can be used to predict metastatic spread and detect early recurrence in BC [35]. Growing body evidence correlating NLR with several biological aspects of BC prompted us to utilize the latest guidelines by the IKWG 2019 stratifying Ki-67 into low ≤ 5%, intermediate 6–29%, and high ≥ 30% grades, and positively correlating them with NLR. A simple, rapid, cheap, and one of the most commonly performed investigation globally, the hemogram, can be used to calculate NLR which can have much profound ramifications than what had been thought for decades. This will go a long way in managing patients of breast cancer.

## Conclusion

The therapeutic and prognostic relevance of Ki-67 which in part reflects the proliferative capabilities of breast cancer can be reinforced by peripheral NLR. Baseline NLR and its dynamics with unpredictable clinical behavior of breast cancer will serve as a minimally invasive personalized marker for early diagnosis, guiding therapy, prognosis, and follow-up of patients.

## Data Availability

All the data has been supplied in the manuscript itself.

## Abbreviations

NLR: Neutrophil to lymphocyte ratio
BC: Breast cancer
IHC: Immunohistochemistry
ER: Estrogen receptor
PR: Progesterone receptor
HER 2/neu: Human epidermal growth factor receptor 2
TNBC: Triple-negative breast cancer
NAC: Neoadjuvant chemotherapy
IKWG: International Ki-67 in Breast Cancer Working Group
HSCs: Hematopoietic stem cells
EDTA: Ethylenediaminetetraacetic acid
H&E: Hematoxylin & Eosin
SBR: Scarff-Bloom-Richardson
SPSS: Statistical Package for the Social Sciences
IBC: Invasive breast cancer
NST: No special type
TNM: Tumor/nodes/metastasis
pCR: Pathological complete response
